# Development of mealtime difficulty scale for older adults with dementia in long-term care facilities

**DOI:** 10.1186/s12877-022-03224-y

**Published:** 2022-06-24

**Authors:** Dukyoo Jung, Eunju Choi, Leeho Yoo, Hyesoon Lee

**Affiliations:** 1 College of Nursing, Ewha Womans University, Seoul, Republic of Korea; 2 Department of Nursing, Semyung University, 65, Semyeong-ro, Jecheon-si, Chungbuk 27136 Republic of Korea

**Keywords:** Aged, Dementia, Mealtime difficulty, Direct care worker, Nursing home

## Abstract

**Background:**

In older patients with dementia, functional dependence on individuals affects their eating behavior, leading to difficulties with meals. In addition to individual factors, several social, cultural, and environmental factors influence mealtime difficulties in older individuals with dementia. Therefore, a measure is required to evaluate the difficulty of eating, considering the different interacting phenomena.

**Methods:**

Mealtime Difficulties Scale for older adults with Dementia (MDSD) was developed through a literature review. A pilot test was undertaken to confirm the meaning of the items and the relevance of mealtime difficulties for older patients with dementia. A panel of six experts examined the content validity of the MDSD. Convenience sampling was used to recruit direct care workers from long-term care facilities, of which 150 were recruited for exploratory factor analysis (EFA) and 208 for confirmatory factor analysis (CFA).

**Results:**

The final version of the MDSD included 19 items, with a Cronbach’s α of 0.91. The EFA identified three factors (“functional,” “caregiving,” and “behavioral”) that account for 54.6% of the total variance. The CFA confirmed the validity of the instrument.

**Conclusions:**

Evidence to substantiate the validity and reliability of MDSD was found. While this tool has limitations in that it does not ensure convergent validity, it can be considered significant as it can assess the mealtime difficulty among older patients with dementia from different perspectives.

**Supplementary Information:**

The online version contains supplementary material available at 10.1186/s12877-022-03224-y.

## Background

Older people with lower cognitive abilities are likely to have low dietary/energy intake [1, 2]. Dementia affects not only memory, mood, social behavior, and communication skills but also appetite, eating, drinking, swallowing, and motor skills in transporting food, all of which may reduce the ability to participate in meals [3]. Meanwhile, hospitalizing older adults with impaired functionality can cause them to experience physiological, psychological, and social changes that affect their dietary behaviors [4]. Additionally, approximately one-third of nursing home residents needed help eating, and residents with severe cognitive impairments and reduced physical abilities were more likely to need direct-care workers to feed them [4, 5].

Nutritional support and feeding might be an essential part of dementia care [6]. Therefore, establishing effective nutrition strategies should be considered a primary goal of nursing care for older adults with dementia and provided to prevent malnutrition in long-term care facilities (LTCF) [4]. Since they have mealtime difficulties, residents with dementia depend on caregivers when they eat on their own and when caregivers feed them. As dementia progresses, the patient may exhibit unusual changes in dietary behavior and eating habits [7]. An appropriate assessment of eating difficulties can help plan preventive intervention strategies for healthy aging or can help identify abnormal behaviors that represent aging-related illnesses [3, 7].

Mealtime difficulty in older adults with dementia has been measured using a few tools such as EdFED [8] and EBS [9]. The Edinburgh Feeding Evaluation in Dementia (EdFED), which was developed to assess eating difficulty in dementia patients, is the most commonly used instrument [10]. EdFED consists of relatively simple items and allows ease of measurement of mealtime difficulty experienced by patients with dementia. However, the questions focus on putting food into the patient’s mouth—for example, “Does the patient indicate a refusal to eat?” and “Does the patient spit food out?” Details related to swallowing or problem behaviors that can occur in patients with dementia even before bringing food to the mouth are not included. Additionally, circumstances that could trigger problem behaviors were not considered. The EBS, on the other hand, only considers patients’ functional disabilities that impede the task and safety of eating [9]. The EBS is a six-item observational checklist that includes eating initiation, maintaining attention, locating all of the food, correct usage of the utensil, safety without choking, and recognition of finishing the meal. Thus, these previous standardized measures only reflect the personal aspects of older adults with dementia through their measurement items and focus only on limited functionality due to cognitive declines in older adults with dementia. However, these instruments fail to consider the behavioral and psychological symptoms of dementia (BPSD), environmental, and interpersonal phenomena relating to that may significantly shape dietary impairments among older adults with dementia [11]. Socio-cultural contexts, such as health and medical policies and services and dietary habits related to food type, can influence dietary behavior during aging [12, 13]. Nursing is in a pivotal position to play a role in contexts that influence eating behavior [9]. A systematic approach makes it easier to solve clinical problems by defining the causes.

“Mealtime difficulties” in dementia patients indicate frustrations or problems occurring during meals. These problems are connected to physical, cognitive, behavioral, social, environmental, and cultural factors [13, 14]. Based on the results of scoping reviews, intrapersonal (cognitive and physical function), interpersonal (attitude toward caregiver meals), and environmental domains (diet) frequently affected mealtime difficulties [11, 15]. Additionally, the scoping review concluded that it is difficult to measure in detail by using just eight or nine items in the existing instruments [11]. Social ecological models (SEM) that apply to mealtime difficulties can optimize the health and well-being of dementia patients. Moreover, SEM can support the development of systematic intervention mechanisms that can change behavior in multiple domains (intrapersonal, interpersonal, environmental, and institutional) [16]. Based on previous studies, we found that a Mealtime Difficulty Scale for older adults with Dementia (MDSD) should be developed including intrapersonal, interpersonal, and environmental domains and the reliability and validity of the tool should be examined.

## Methods

### Design

This methodological study based on the measurement development procedure suggested by DeVellis [17] was conducted to develop and validate the mealtime difficulty scale for older adults with dementia in LTC; then, its validity and reliability were tested.

### Participants

Data were gathered from November through December 2021. A convenience sample of direct care workers (nurses and caregivers) who worked at the LTCF for over 6 months and had experience caring for older adults with dementia was used. We explained the research purpose and obtained the LTCF directors’ approval. Recruitment notices were posted on the facilities ‘s bulletin board. The number of participants for exploratory factor analysis (EFA) [18] should be at least 145, which is 5 times the number of questions (29) because the number of participants should be 5 to 10 times higher than the number of questions. With a 10% dropout rate, data were gathered from 160 participants. Secondly, for confirmatory factor analysis (CFA), the number of participants should be at least 200 [19]; the total number of participants was 220, given the 10% dropout rate. So the total number of participants included in the EFA and CFA analysis was 380. After excluding 10 participants from the first and 12 from the second data collection due to inadequate response, 358 participants were identified: 150 for EFA and 208 for CFA.

### Phase 1. Developing MDSD

#### Item composition

We reviewed articles on eating difficulties among older adults with dementia in LTCF in our scoping review and developed items using the final 39 articles. In previous studies [11], the initial total number of items was 31. The developed MDSD was designed to allow direct care providers to answer the questionnaire for the older adult with dementia they mainly care for. Each item was evaluated on a 5-point Likert scale. The higher the score, the greater the mealtime difficulty.

#### Content validity

To verify the content validity of the completed MDSD, a group consisting of three field experts operating nursing facilities with > 10 years of nursing experience and three geriatric nursing professors was formed. The group was asked to rate each scale item using a 4-point Likert scale: 1 (Not relevant), 2 (Needs modification), 3 (Some modification required), and 4 (Relevant and concise). After verifying the validity, the Item Content Validity Index (I-CVI) and the Average of Content Validity Index for Scale (S-CVI/Ave) were determined. Content validity is secured when the I-CVI score is .78 or higher and when the S-CVI/Ave score is .90 or higher [20].

#### Preliminary investigation

After content validity analysis, a preliminary survey was conducted with three nurses and three caregivers who met the selection criteria. On this basis, the researchers discussed and revised any items that were difficult to understand due to terminology or a large amount of content. This revision was the final step in drafting the 29-item MDSD.

### Phase 2. MDSD validation

#### Data collection and ethics consideration

Prior to conducting the study, the study obtained approval from the IRB (Institutional Review Board) (ewha-202,111-0010-02). Data were collected between November and December 2021 using convenience sampling of 12 LTCFs, after explaining the purpose of this study and obtaining consent from all subjects for data collection.

#### Instrument

The EBS developed by Tully et al. [9] and translated by Lee & Song [21] was used to verify the criterion validity of the tool for difficulty in eating. The lower the total score, the lower the ability to eat and the higher the dependency [22]. A score of 12 or higher was classified as mild dependence, a score of 6–11 as moderate, and a score of 5 or less as severe [22]. At the time the tool was developed, Tully et al. [9] found the inter-rater reliability was 0.959 and the Cronbach’s alpha was 0.78 in the Lee & Song study [21].

#### Validity test

This study adopted the SPSS statistics 26.0 program and AMOS 23.0 program for data analysis. The demographic characteristics of the subjects were studied using frequency and percentage methods. Item analysis was performed based on item means, standard deviations, skewness < 3, and kurtosis < 7. Next, the item-total correlation was examined to determine the contribution each item made. Item-total correlation coefficient below 0.30 were evaluated as having low discriminatory power [23] and were therefore considered for deletion.

EFA and CFA were applied for the construct validity test of this tool. For EFA, principal component analysis was used, and a varimax rotation method was adopted. An eigenvalue of 1.0 or more, a total cumulative variance of 50% or more, and a factor loading of 0.30 or more [23] were used to determine the number of factors for item selection. The CFA was carried out to confirm the structural conformity of the derived factors and the following was applied: normed χ2 (CMIN/df) < 3.00, goodness of fit index (GFI) ≥ 0.90, root mean square error of approximation (RMSEA) < 0.05–0.08, comparative fit index (CFI) ≥ 0.90, Tucker-Lewis Index (TLI) ≥ 0.90, incremental fit index (IFI) ≥ 0.90 [24] and standardized root mean residual (SRMR) < 0.08 [25].

For the convergent validity, the construct reliability (CR) of 0.70 or more was applied [24]. An item’s discriminant validity was determined by not considering a confidence interval of 1.0 for the correlation coefficient between factors [26]. The EBS developed by Tully et al. [9] and translated by Lee & Song [21] was used to establish the criterion validity of MDSD, and correlation analysis was performed using Pearson’s correlation coefficient. The corrected item-total score correlation (ITC) was adopted to test the reliability of the tool and the Cronbach’s alpha coefficient was used to analyze internal consistency. The intraclass correlation coefficient (ICC) for each factor was confirmed.

## Results

### Demographic characteristics

Table 1 shows the general characteristics of the caregivers in LTCF. A total of 358 caregivers in LTCF were studied, the majority of whom were female (*n* = 343,95.8%) with an average age of 58.52 ± 6.68 years. As far as the level of education is concerned, 232 subjects (64.8%) were high school graduates. The majority of subjects, 326 (91.1%), worked as caregivers. The average employment period was 3.18 ± 2.36 years, and the average employment period to present employment was 3.24 ± 3.13. The average number of older adults per caregiver in LTCF was 6.89 ± 7.74. There were 257 caregivers working in private facilities (71.8%), and 169 caregivers working in facilities with 75 to 100 beds constituted the largest proportion (47.2%). The number of caregivers working 8 hour shifts was 203 (56.7%), accounting for the largest proportion of subjects. Additionally, 314 subjects (87.7%) had experience in education on dietary behavior for older adults with dementia.

**Table 1:** Demographic Characteristics of Participants (*N* = 358)

Characteristics	Categories	Total (*N* = 358)	Participants for EFA (*N* = 150)	Participants for CFA (*N* = 208)
		n (%) or M ± SD	n (%) or M ± SD	n (%) or M ± SD
Age		58.52 ± 6.68	58.65 ± 6.90	58.43 ± 6.54
Gender	Male	15 (4.2)	10 (6.7)	5 (2.4)
	Female	343 (95.8)	140 (93.3)	203 (97.6)
Level of education	< Middle school	53 (14.8)	25 (16.7)	28 (13.4)
	High school	232 (64.8)	93 (62.0)	139 (66.8)
	≥ University	73 (20.4)	32 (21.3)	41 (19.7)
License	Nurse	32 (8.9)	14 (9.3)	18 (8.7)
	Personal care assistant	326 (91.1)	136 (90.7)	190 (91.3)
Total work experience (Years)		3.18 ± 2.36	3.26 ± 2.31	3.13 ± 2.41
Number of residents to be managed		6.89 ± 7.74	7.03 ± 7.23	6.78 ± 8.12
Facility type	Private	257 (71.8)	133 (88.7)	124 (59.6)
	Corporate	101 (28.2)	17 (11.3)	84 (41.4)
Facility size	< 25 beds	15 (4.2)	11 (7.3)	4 (1.9)
	25–50 beds	51 (14.2)	8 (5.3)	43 (20.7)
	50–75 beds	57 (15.9)	20 (13.3)	37 (17.8)
	75–100 beds	169 (47.2)	99 (66.0)	70 (33.7)
	> 100–200 beds	66 (18.4)	12 (8.0)	54 (26.0)
Duty	Fixed	31 (8.7)	19 (12.7)	12 (5.8)
	8 hour shift	203 (56.7)	67 (44.7)	136 (65.4)
	12 hour shift	118 (33.0)	60 (40.0)	58 (27.9)
	24 hour	6 (1.7)	4 (2.7)	2 (1.0)
Eating behavior education experience	Yes	314 (87.7)	126 (84.0)	188 (90.4)
	No	44 (12.3)	24 (16.0)	20 (9.6)

### Validity

#### Content validity

I-CVI and S-CVI/Ave were calculated for content validity. The content validity of 31 items was verified (Table 2). Based on the results, these two items with a CVI of 0.66, “the older adults with severe dementia cannot swallow due to abnormalities in vital signs” and “the older adults with dementia do not eat for more than 1 minute after starting a meal,” were omitted. With the exception of the two items deleted, the I-CVI of all 29 items was more than 0.8, while the S-CVI/Ave was 0.96. Additionally, the opinions of six experts were partially altered and reflected in the items to help the subjects better understand the items and improve the flow of the sentences.

**Table 2:** Content Validity

Initial No.	No.	Item	I-CVI
1	1	Refuses to eat food	1
2	2	Spits out the food	1
3	3	Once food is in the mouth, food dribbles out from the mouth	1
4	4	Does not initiate swallowing	1
5	5	Does not chew food and continuously holds it in the mouth	1
6	6	Bites the utensils when food is offered	1
7	7	Uses hands to eat	1
8	8	Unable to successfully pick up food with utensils	1
9	9	Plays with food but does not eat it	1
10	Delete	In case of severe dementia, swallowing is not possible due to abnormal vital signs.	0.66
11	10	Eats something other than food	0.83
12	Delete	Does not eat for more than 1 minute after starting a meal	0.66
13	11	Distracted from eating	1
14	12	Forgets when they last ate and eats too much food	1
15	13	Does not consume a variety of food and eats one type of food	0.83
16	14	Frequently chokes or gags on food	1
17	15	Unable to eat food because of pain	1
18	16	Unable to maintain posture while eating	0.83
19	17	Needs care from the feeder to finish the meal	1
20	18	Eats alone	1
21	19	Negative behavior (e.g., swearing, throwing food) toward the caregiver	1
22	20	Needs active encouragement (e.g., compliments, suggestions) from the caregiver while eating	0.83
23	21	No interaction with caregiver during meals	1
24	22	Receives sufficient meal support from caregiver	1
25	23	Lack of caregivers.	1
26	24	Food preferences are not reflected in their diet	1
27	25	Meal types (e.g., regular, liquid, soft) are provided based on swallowing function	1
28	26	Appropriate tableware (e.g., cups with straws) is provided	1
29	27	The height of the furniture (e.g., table, chair, bed table) used for dining is appropriate	1
30	28	Eats in unpleasant environments (e.g., dark lighting, noisy spaces, bad smelling places).	1
31	29	Unable to finish food within the given timeframe	1
	S-CVI/Ave		0.96

*I-CVI* Item-content validity index, *S-CVI/Ave* Average of item-content validity index

#### Preliminary survey

Six nurses and caregivers with experience with dementia patients in a care facility participated in the preliminary survey. The average time taken to complete the survey was 5.05 ± 2.01 minutes. The preliminary survey found an average of 3.14 points for the comprehension of the questions, and an average of 3.50 points for the relevance of the number of questions. The average time taken to fill out the survey was 9.40 minutes. Some items were revised to reflect participants’ views on the difficulty or ambiguity of those items.

#### Item analysis

As a first step in the item analysis, the mean, standard deviation, skewness, and kurtosis of each item were examined to see if the data collected was suitable for analysis. To test multivariate normality for structural equation model analysis, item 28 was removed as it did not meet the criteria, had an absolute skewness value below 3, and an absolute kurtosis of below 7.

The correlation coefficient between each item and the questionnaire was computed. 8 items did not meet the criterion of item-total correlations were considered to delete. However, two items with almost no change in alpha when the item was deleted were maintained. And 6 items were excluded. Therefore, the item-total correlation coefficient of the 22 items was 0.286–0.646 [27].

#### Construct validity

The Kaiser-Meyer-Olkin (KMO) test was conducted to determine the suitability of the data for EFA: The value was 0.886. Bartlett’s sphericity test was performed to determine if the correlation coefficient matrix was appropriate for factor analysis. The test value was x^2^ = 1549.30 (*p* < 0.001), confirming that this data was adequate. Principal component analysis and orthogonal rotation were applied to factor analysis. The eigenvalues in the principal component analysis indicated that the communality between the 22 items was 0.312–0.741. Items with a communality of 0.4 or less were recommended for removal [28], but item 29 with a communality of 0.312 was not omitted because the researchers decided after discussion that the item was necessary to measure eating difficulties. For number of factors, we reflected factors with eigenvalue equal to or greater than 1.0 according to Kaiser’s recommendation [29] and referred to the scree plot. The number of factors was determined based on the cumulative variance ratio and the factors’ interpretability. Additionally, only factors with three or more items per factor were considered significant for the analysis [30]. The EFA used the criterion that a factor loading of an item greater than 0.30 was significant. However, there were no factor loadings below 0.30 [31] in this study. In the first EFA, there were four factors with an eigenvalue of 1.0 or higher. However, when analyzed with four factors, one factor had two variables. As factors containing at least three variables are viable [32], the number of retention factors needed adjustment. The analysis was conducted using three factors that met the criteria, namely, the cumulative variance ratio of 50% or greater [33]. Items 10, 24, and 29 did not meet the criteria of communality (0.4 or less). Item 24 was deleted, on the other hand, items 10 and 29 were retained as we decided both were necessary to measure eating problems in older adults with dementia. There were three items with a factor loading of 0.30 or more in two factors. Item 14 (the older adults with dementia often have food go down the wrong pipe) was cross-loaded on Factors 1 and 2. Item 16 (it is difficult for the older adults with dementia to maintain posture while eating) was cross-loaded on Factors 1 and 2, as was item 29 (the older adults with dementia take a longer time to eat). If a factor loading of 0.3 or higher is shown in two or more factors, removal of the item must be considered given the difference between the content and the factor load of the item [34]. Items 14 and 16 were therefore appropriate for Factor 1 and item 29 for Factor 2. Also, having food stuck, maintaining posture while eating, and taking longer to eat were symptoms necessary to determine eating problems in the older adults with dementia. Therefore, they were retained for further analysis. The explanatory power for the last three factors was 54.62%. As all factor loading ranged from 0.38 to 0.83, they also met the criterion that the value should be above 0.3 and not close to 1.0 [35].

To assess the model fit of the three factors and 21 items derived from the EFA, we performed CFA on 208 new caregivers in LTCF. Standardized regression weights of the eight items (1, 8, 11, 16, 17, 20, 23, and 29) were below 0.5. Although the weight should be 0.5 or more to be appropriate [36], it was decided, following discussion among the researchers, to leave the six items (1, 11, 16, 20, 23, and 29) that were deemed essential to complete the content of the factors. Model identification was not a problem even after removing the items because each latent variable had three or more items. The results were Chi-square = 362.21 (*p* < .001), CMIN/df = 2.43, GFI = 0.84, SRMR = 0.07, CFI = 0.86, TLI = 0.84, IFI = 0.86, and RMSEA = 0.07, indicating that CFI, TLI, and IFI were less than 0.9. The study assumed a correlation between item errors by referencing the correction index to explore the possibility of improving the model. The model was finally modified by assuming that the covariance of errors with a correction index of four or more among the errors of the items belonged to the same factor [26]. The results were Chi-square = 231.71 (*p* < .001), CMIN/df = 1.67, GFI = 0.90, SRMR = 0.06, CFI = 0.94, TLI = 0.92, IFI = 0.94, and RMSEA = 0.06. Therefore, it has been proven that the model fits the data.

Convergent and discriminant validity were assessed to check the validity of the items constituting the model. Convergent validity was obtained with functional and behavioral factors in the CR range of 0.77–0.86, but convergent validity was not obtained for caregiving factors. Additionally, the correlation coefficient between factors was 0.718–0.762, and the confidence interval of the correlation coefficient between factors did not include 1.0, so discriminant validity was secured (Table 3).

**Table 3:** Factor analysis of the MDSD

Category	Item no.	Exploratory factor analysis				Confirmatory Factor analysis		
		Communality	Factor structure			CR	*p*-value	FL
			1	2	3			
Functional factor	3	0.66	**.770**	.142	.213	–	–	–
	5	0.70	**.761**	.126	.308	8.79	<.001	0.695
	4	0.61	**.761**	.182	.031	9.33	<.001	0.67
	2	0.59	**.722**	.234	.104	11.06	<.001	0.72
	1	0.48	**.668**	.128	.128	5.84	<.001	0.691
	11	0.57	**.648**	.245	.296	6.46	<.001	0.441
	15	0.49	**.622**	.315	−.090	9.32	<.001	0.488
	6	0.59	**.621**	.071	.446	10.38	<.001	0.717
	8	0.42	**.484**	.315	.292	Delete	Delete	Delete
	14	0.54	**.509**	.527	.041	7.69	<.001	0.58
	16	0.51	**.470**	.488	.215	6.52	<.001	0.49
Caregiving factor	20	0.73	.222	**.822**	−.033	–	–	0.408
	23	0.55	.082	**.684**	.274	3.45	<.001	0.357
	17	0.65	.278	**.659**	.365	Delete	Delete	Delete
	19	0.50	.137	**.613**	.317	4.54	<.001	0.69
	29	0.31	.326	**.381**	.242	2.62	.009	0.244
Behavioral factor	7	0.70	.000	.013	**.834**	–	**–**	0.75
	12	0.51	.113	.268	**.654**	7.53	<.001	0.557
	13	0.47	.270	.283	**.566**	7.09	<.001	0.525
	9	0.60	.525	.183	**.535**	10.76	<.001	0.793
	10	0.33	.220	.252	**.470**	10.28	<.001	0.755
	Explained (%)	25.20	15.55	13.88	Model Fit	*x*^*2*^ = 231.71 (*p* < .001), *x*^*2*^/df = 1.67, GFI = 0.90, CFI = 0.94, TLI = 0.92, IFI = 0.94, SRMR = 0.06, RMSEA =0.06		
	Cumulative (%)	25.20	40.75	54.63				

*CR* Critical ratio, *FL* Standardized factor loading, *GFI* Goodness of fit index, *CFI* Comparative fit index, *TLI* Tucker-Lewis index, *IFI* Incremental fit index, *SRMR* Standardized root mean square residual, *RMSEA* Root mean ssquare error of approximation

#### Criterion validity

This research used an EBS tool, developed by Tully et al. [9] and translated by Lee and Song [21] to identify the criterion validity of MDSD; it was a practical method to evaluate the motor difficulties of the older adults with dementia. Pearson’s correlation was measured across all 19 MDSD items (Additional file 1). It was found to be negatively correlated with the Korean version of EBS (*r* = − 0.15, *p* = 0.03). Therefore, there is a positive correlation with concepts similar to this tool, and criterion validity has been established.

#### Reliability

Internal consistency verification of questions with identified construct validity showed a Cronbach’s α value of 0.91. The Cronbach’s α for each factor are: 0.90 for Factor 1 (Functional factors), 0.80 for Factor 2 (Behavioral factor), and 0.64 for Factor 3 (Caregiving factors). Generally, the internal consistency is considered appropriate if it is 0.6–0.70 or greater [37, 38]. Thus, internal consistency was established.

## Discussion

The MDSD was developed as part of this study to address the mealtime difficulties of older adults with dementia in the long-term care facilities in South Korea while taking into account external characteristics.

Based on the previous scoping review [11], three domains (intrapersonal, interpersonal, and environmental) with 31 items were identified early in the development of the measure. When examining the construct validity of EFA and then CFA, the environmental domains were eliminated, and the intrapersonal domain was divided into functional and behavioral domains. Therefore, the MDSD ultimately consisted of two domains (intrapersonal and interpersonal), three factors (functional, caregiving, behavioral), and 19 items.

In the previous study, the environmental factor was one of the important aspects in supporting mealtime difficulties among older adults with dementia. Older adults with dementia who are easily distracted by the cognitive environment benefit from eating in public places and being provided with an appropriate meal type. Because social interactions and appropriate environments (light, sound etc.) affect mealtime difficulty, eating in communal areas makes older adults less dependent than eating in individual rooms [22]. Soft and liquid meals increasingly deteriorate eating ability and increase mealtime difficulty [39]. Therefore, the initial items of the MDSD included environmental components in the long-term care facilities such as the height of the table, provision of preferred food, various types of meals, appropriate dishes, comfortable environment for eating (noise, smell, lighting etc.), sufficient mealtime, and a ratio of staff between direct care workers and residents. However, a couple of items about the environment were removed and some items were classified under the caregiving factor in the final version of the MDSD. We assumed that the perception of the environment in long-term care facilities might differ among the direct care workers who responded to the original MDSD.

The functional factor of MDSD contained 10 items. In this factor, the items consisted mainly of decreased physical function and cognitive deterioration of residents. The items of this functional factor are similar to EdFED, which contained dietary difficulties such as refusal to eat, spitting, and inability to swallow [8]. The behavioral factor for MDSD was abnormal behavior with impaired cognitive behavior and contained five items. The items are related to BPSD, which may lead to mealtime difficulties. Direct care workers can develop a meal assistance plan based on the evaluation of the function and behaviors of residents with dementia as a part of this MDSD. Additionally, the four items in the caregiving factor. This factor included how the caregiver interacted with residents with dementia during meals. Mealtime difficulties could mainly come from functional and behavioral factors and they would deteriorate as dementia worsens. The caregiving factor would be a modifiable factor that can improve or worsen the mealtime difficulties and MDSD could assist direct care workers in supporting older adults with dementia. More specifically, the development of the MDSD can facilitate the evaluation of various aspects of mealtime difficulty and identify their causal factors. Identifying the causes of mealtime difficulties will allow caregivers to more effectively solve them.

### Limitations

Since most environmental items in mealtime difficulties were removed and classified as caregiving factors, this measurement did not sufficiently reflect the environmental aspects of mealtime difficulty; this limitation should be considered when using the MDSD. Additionally, there was a lack of evidence of convergent validity. Therefore, the validity of MDSD should be examined through a replicated study. This measure does not bring the level of mealtime difficulties necessary for analyzing and discriminating the level of food support. Therefore, for further investigation, further analysis, including item analysis, is recommended.

## Conclusions

There was evidence to support the validity and reliability of MDSD. Although this MDSD has limitations in that convergent validity was not confirmed, the study is important in that it has developed a measure that can assess the mealtime difficulty among older adults with dementia from various perspectives. Notably, such assessments are necessary to design interventions in mealtime difficulty tailored to its cause.

## Supplementary Information

**Additional file 1.** Description of data: Final version of the Mealtime Difficulty Scale for Older Adults with Dementia in Long-term care facilities

## Data Availability

The datasets used and/or analyzed during the current study are available from the corresponding author upon reasonable request.

## Abbreviations

MDSD: Mealtime difficulties Scale for older adults with dementia
BPSD: Behavioral & psychological symptoms of dementia
SEMs: Social ecological models
CFA: Confirmatory factor analysis
EFA: Exploratory factor analysis
AVE: Average variance extracted
CFI: Comparative fit index
GFI: Goodness of fit index
RMSEA: Root mean square error of approximation
CFA: Comparative fit index
TLI: Tucker-Lewis index
IFI: Incremental fit index
SRMR: Standardized root mean residual
ITC: Item-total score correlation
ICC: The intraclass correlation coefficient
CR: Construct reliability
LTCF: Long-term care facilities
EBS: Eating behavior scale
NH: Nursing homes
I-CVI: Item content validity index
S-CVI: The scale content validity index
KMO: Kaiser-Meyer-Olkin
EdFED: Edinburgh Feeding Evaluation in Dementia
