# Supplementary material for: Development of mealtime difficulty scale for older adults with dementia in long-term care facilities
Source: BMC Geriatr. 2022 Jun 24;22:518. doi: 10.1186/s12877-022-03224-y (PMC9229116; doi:10.1186/s12877-022-03224-y)
Supplement: Supplementary file 1 — Additional file 1. Description of data: Final version of the Mealtime Difficulty Scale for Older Adults with Dementia in Long-term care facilities [file 12877_2022_3224_MOESM1_ESM.docx]

Appendix1. Final version of the Mealtime Difficulty Scale for Older Adults with Dementia in Long-term care facilities (MDSD)

| Q1 | Once food is in the mouth, food dribbles out from the mouth |
| --- | --- |
| Q2 | Does not chew food and continuously holds in the mouth |
| Q3 | Does not initiate swallowing |
| Q4 | Spits out the food |
| Q5 | Refuses to eat food |
| Q6 | Distracted from eating |
| Q7 | Unable to eat food because of pain |
| Q8 | Bites the utensils when food is offered |
| Q9 | Frequently chokes or gags on food |
| Q10 | Unable to maintain posture while eating |
| Q11 | Uses hands to feed self |
| Q12 | Has forgotten when they last ate and eats too much food |
| Q13 | Does not consume a variety of food and eats one type of food |
| Q14 | Plays with food but does not eat it |
| Q15 | Eats something else other than food |
| Q16 | Needs active encouragement (compliment, suggestion) from the caregiver while eating |
| Q17 | Lack of caregivers |
| Q18 | Negative behavior (swearing, throwing food) toward the caregiver |
| Q19 | Unable to finish food within the given timeframe |

The subject of all questions is 'Older adults with dementia'.
